# Heat-related mortality in Frankfurt am Main, Germany, from 2000 to 2023

**DOI:** 10.3205/dgkh000477

**Published:** 2024-04-30

**Authors:** Ursel Heudorf, Bernd Kowall, Eugen Domann, Katrin Steul

**Affiliations:** 1 Institute of Hygiene and Environmental Medicine, Justus Liebig University, Giessen, Germany; 2 Institute for Medical Informatics, Biometry and Epidemiology, University Hospital Essen, Germany; 3 Institute of Occupational, Social and Environmental Medicine, University Medical Centre of the Johannes Gutenberg University, Mainz, Germany

**Keywords:** heat, heat day, heat week, heat warning, heatwave, heat-associated mortality

## Abstract

**Background:**

The major heatwave in Europe in August 2003 resulted in 70,000 excess deaths. In Frankfurt am Main, a city with 767,000 inhabitants in the south-west of Germany, around 200 more people died in August 2003 than expected. Soon afterwards, the city introduced adaptation measures to prevent heat-related health problems and subsequently established further mitigation measures to limit climate change. Frankfurt is rated as being one of the cities in Germany to have implemented the best climate adaptation and mitigation measures. This study addressed the following questions: is there already a downward trend in mortality from heat and can this be attributed to the measures taken?

**Materials and methods:**

The age-standardized mortality rate (ASR) was calculated for the months of June to August and for calendar weeks 23 to 34 of the individual years on the basis of population data and deaths of the inhabitants of Frankfurt am Main for the years 2000 to 2023. This was related to the meteorological data from the Frankfurt measuring station of the German National Meteorological Service. For four different heat exposure indicators (heat days, days in heat weeks, days in heatwaves and days with heat warnings), the incidence rate (death cases per 1 million person days) (IR) was calculated for days with and without exposure, and the incidence rate difference and the incidence rate ratio (IRR) were estimated to compare days with vs days without exposure.

**Results:**

Over the years, the mean daily temperatures tended to increase, and the standardized mortality rate decreased. An increase in ASR was observed during heatwaves up to 2015, but no longer in the later ones. In the summer of 2003, the incidence rate was 16.0 (95% confidence interval (CI) 12.2–19.9) per 1 million person days greater on heat days than on days not classified as heat days, and the corresponding incidence rate ratio was 1.64 (95% CI 1.48–1.82). Although the weather data for the summers of 2018 and 2022 were comparable with the record-breaking heat summer of 2003, the incidence rate differences (2018: 3.8, 95% CI 0.9–6.7; 2022: 2.3, 95% CI –0.3–4.9) and the IRR (2018: 1.20, 95% CI 1.05–1.37; 2022: 1.12, 95% CI 0.99–1.26) were considerably lower. Similar results were also obtained when comparing mortality in heat weeks and heatwaves as well as on days with heat warnings.

**Discussion:**

In summary, our study in Frankfurt am Main not only showed a decrease in heat-related mortality in the population as a whole over the years, but also a decrease in excess mortality during various heat periods (day, week, wave, warning), especially in comparison with the years with very high heat stress and drought (2003, 2018 and 2022). However, whether this development represents success of the intensive prevention measures that have been implemented in the city for years or merely describes a general trend cannot be answered with certainty by the present study. To answer this question, a comparative study should be carried out in various municipalities in the Rhine-Main region with different levels of intensity in dealing with the heat problem.

## Zusammenfassung

**Hintergrund:**

Bei der großen Hitzewelle im August 2003 in Europa kam es zu einer Übersterblichkeit von 70.000 Menschen. Allein in Frankfurt am Main verstarben im August 2003 ca. 200 Menschen mehr als erwartet. In der Stadt wurden bald darauf Adaptationsmaßnahmen zur Prävention hitzeassoziierter Gesundheitsstörungen eingeleitet und in der Folge weitere Maßnahmen zur Mitigierung der Klimafolgen etabliert. Frankfurt zählt in Deutschland zu den Städten mit den besten Klimaadaptations- und Mitigierungsmaßnahmen. Nachfolgend wird den Fragen nachgegangen, ob es einen bereits abnehmenden Trend der Sterblichkeit bei Hitze gibt und ob dieser auf die ergriffenen Maßnahmen zurückgeführt werden kann.

**Material und Methode:**

Anhand der Bevölkerungsdaten sowie der Sterbefälle der Bewohner von Frankfurt am Main der Jahre 2000 bis 2023 wurden die alters-standardisierte Mortalitätsrate (ASR) für die Monate Juni–August und für die Kalenderwochen 23–34 der einzelnen Jahre berechnet und mit den Wetterdaten der Messstelle Frankfurt des Deutschen Wetterdienstes verglichen. Für vier verschiedene Hitze-Expositions-Indikatoren (Hitzetag, Tage in Hitzewochen, Tage in Hitzewellen und Tage mit Hitzewarnungen) wurden jeweils für Tage mit und ohne Exposition die Inzidenzrate pro Million Personentage und die Differenz der Inzidenzraten sowie der Quotient (Inzidenzraten Ratio IRR) für Tage mit und ohne Exposition berechnet.

**Ergebnisse:**

Über die Jahre nahmen die mittleren Tagestemperaturen tendenziell zu, die alters-standardisierte Mortalitätsrate ab. In den Hitzewellen bis 2015 war eine Zunahme der ASR zu beobachten, in den späteren Jahren nicht mehr. Im Sommer 2003 hatte die Inzidenzrate pro 1 Million Personentage an Hitzetagen um 16,0 (95% Kofidenzintervall (KI) 12,2–19,9) zugenommen und die Inzidenzraten Ratio IRR 1,64 (95% KI 1,48–1,82) betragen. Obwohl die Wetterdaten der Sommer der Jahre 2018 und 2022 mit dem „Hitzesommer“ 2003 vergleichbar waren, waren die Zunahme der IR (2018: 3,8, 95% KI 0,9–6,7; 2022: 2,3, 95% KI –0,3–4,9) und die IRR (2018: 1,20, 95% KI 1,05–1,37; 2022: 1,12, 95% KI 0,99–1,26) deutlich geringer. Ähnliche Ergebnisse wurden auch beim Vergleich der Mortalität in Hitzewochen und Hitzewellen sowie an Tagen mit Hitzewarnungen erhalten.

**Diskussion:**

Zusammengefasst konnte in unserer Untersuchung in Frankfurt am Main nicht nur eine Abnahme der Hitzemortalität im Bevölkerungsbezug insgesamt über die Jahre, sondern auch eine Abnahme der Übersterblichkeit während verschiedener Hitzeperioden (Tag, Woche, Welle, Warnung) gezeigt werden, insbesondere auch im Vergleich zwischen den Jahren mit sehr hoher Hitzebelastung und Trockenheit (2003, 2018 und 2022). Ob diese Entwicklung einen Erfolg der bereits seit Jahren in der Stadt intensiv durchgeführten Präventionsmaßnahmen darstellt oder lediglich einen generellen Trend beschreibt, kann durch die vorliegende Untersuchung jedoch nicht sicher beantwortet werden. Hierzu sollte eine vergleichende Untersuchung in verschiedenen Kommunen mit unterschiedlich intensiver Bearbeitung der Hitzeproblematik in der Rhein-Main-Region angestrebt werden.

## Introduction

In August 2003, Europe, particularly France, but also other countries as well as southern Germany, was hit by the worst heatwave since 1950 [1]. Around 70,000 heat-related deaths occurred across Europe [2]. For Germany, estimates of the resulting deaths are around 10,000 [3]. The German National Meteorological Service set up a heat warning system in 2005 [4]. Based on the recommendations of the World Health Organization on heat-health action plans [5], the German Adaptation Strategy [6] was developed and “Recommendations for the development of heat action plans” [7] were published. Finally, in 2020, the health ministers of the German federal states adopted a resolution according to which all municipalities should draw up a heat-health action plan within 5 years and stakeholders from the health sector were to be involved [8]. Evaluations to date have shown that although elements of these heat-health action plans have been introduced in many regions and municipalities, overall a lack of coordinated heat action plans, a lack of evaluation of the measures as well as a lack of systematic monitoring of mortality and morbidity was found [9], [10].

In Frankfurt am Main alone, a city with 767,000 inhabitants situated in the Rhine-Main region in south-west Germany, circa 200 more people than expected died in the extreme heatwave of 2003, around half of whom were residents of nursing homes for the elderly [11]. As a result, the state of Hesse set up a working group on heat prevention, which included a representative of the Frankfurt health department. Among other things, under the leadership of the Hessian Care and Nursing Super-vision Department, a guideline for care facilities for the elderly and disabled was developed to prevent heat-related damage to the health of those in need of care, which was later praised as a lighthouse project [12]. Moreover, Hesse was one of the first federal states in Germany to establish a heat warning system by the German National Meteorological Service in 2005. In Frankfurt, intensive training courses for care facilities and physicians were carried out as early as 2004 [13], and further measures for the prevention of heat-related health consequences were introduced based on the recommendations of the World Health Organization [14]. For example, recommendations on appropriate behavior during hot weather have been developed not only for care facilities for the elderly, but also for the general population and especially for schools and daycare centers. Alongside heat warnings issued by the German National Meteorological Service as well as during other heat periods, the public health department carried out intensive public relations work. As early as 2006, the municipal climate change group was established in Frankfurt under the leadership of the Frankfurt Environmental Agency, in which representatives from the Public Health Department, the Fire and Rescue Department, the Building and Civil Engineering Department, the Green Spaces Department, the Department for Roads and Transportation, and the Department for Urban Drainage worked together to develop the Frankfurt Climate Change Adaptation Strategy (2016), which was updated in 2022 [15]. Finally, in 2023, the Frankfurt climate change action plan was drawn up that focuses not only on heat, but also on drought, heavy rainfall, flooding, etc. [16].

Following the first description of excess mortality during the 2003 heatwave [17], the city’s health department has regularly monitored the mortality of residents in Frankfurt and, since 2015, also the morbidity during the summer months based on ambulance transports in Frankfurt [11], [18], [19], [20], [21]. The last publication on heat-associated mortality examined the effects of the 2015 heatwave in comparison with the major heatwave in 2003 [18]. The present study updates the mortality monitoring and thus also covers the hottest summers of recent years, in particular 2018 and 2022. The study examines whether and how heat-related mortality has changed in Frankfurt, and whether this can be attributed to the adaptation measures established. For the first time, not only heatwaves are considered, but also other exposure indicators, such as heat days, heat weeks and heat warnings.

## Material and method

Meteorological data, i.e., daily mean, minimum and maximum temperatures, and data on relative humidity for the summer months of June to August from 2000 to 2023 [22] as well as the days with heat warnings for Frankfurt am Main [23] were obtained from the homepage of the German National Meteorological Service.

The deaths of residents of Frankfurt am Main in the summer months (June to August) of 2000 to 2023 were obtained from the Hessian State Office for Health and Care (for each death, the date of death and age of the deceased were provided).

The number of residents in Frankfurt am Main was taken from the statistical yearbooks of the Citizens’ Office for Statistics and Elections [24]. Until 2021, the mid-year population was estimated from the mean values of the population data given therein at the end of the respective year and the previous year. For 2022 and 2023, the mid-year population was obtained directly from the Residents’ Registration Office.

This was used to calculate the weather data (mean values) and the deaths (totals) for the summer months, the individual months and calendar weeks 23 to 34, as well as the age-standardized mortality rates (ASR), using the population data of Frankfurt for the year 2000 as the reference. 

Further evaluations were carried out with regard to days with heat warnings from the German National Meteorological Service [4], heat days, defined as days with a maximum temperature ≥32°C, heat weeks, defined as weeks with a weekly average temperature ≥20°C, heatwaves, defined as ≥5 heat days (≥32°C) in a row. The incidence rates per 1 million person days (IR) on days with the respective exposure were compared with the incidence rates without this exposure, both as differences (incidence rates on days with definition – incidence rates on days without definition, 95% confidence interval) and as incidence rate ratio (IR on days with definition/IR on days without definition, 95% confidence interval).

## Results

**Figure 1:**
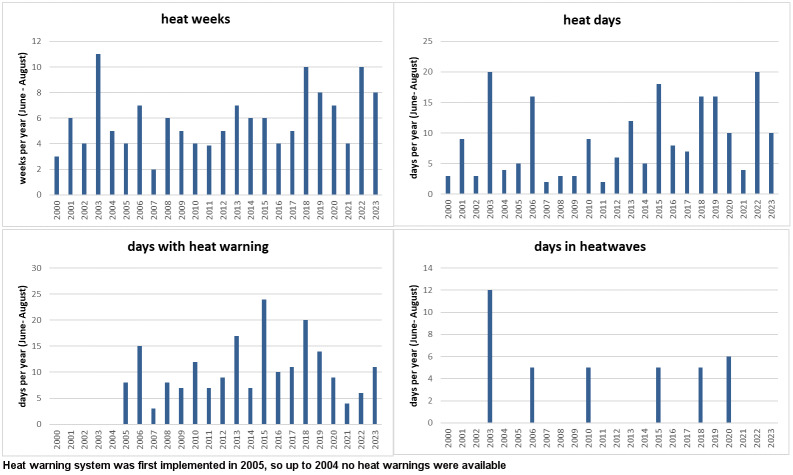
Heat weeks, heat days, days during heat warnings and heatwaves in Frankfurt am Main, Germany, June to August 2000–2023 (German National Meteorological Service measuring station in Frankfurt am Main, Germany)

Table 1 (Tab. 1) shows the meteorological data for the summer months and the number of heat weeks, heat days, days with heat warnings and heatwaves for the years 2000 to 2023. The highest mean values for daily mean, minimum and maximum temperatures were measured in 2003, followed by 2022 and 2018. These three years were not only the warmest, but also the driest years, with the lowest mean relative humidity and precipitation levels in Frankfurt am Main. 2003 was also the year with the most heat weeks (n=11) and the longest heatwave. There were 10 heat weeks in both 2018 and 2022. In 2018, there was a 5-day heatwave as defined above, a total of 16 heat days and 20 days with a heat warning. In 2022, the maximum temperature rose to ≥32°C on 22 days, but no heatwave as defined above occurred, and the number of 6 heat-warning days was comparatively low. In addition, heatwaves were also observed in 2006, 2010, 2015 and 2020. 2015 was the year with the most heat-warning days (Table 1 (Tab. 1) and Figure 1 (Fig. 1)).

The city’s population increased by 25% between 2000 and summer 2023 (from 613,886 to 767,434), and the group aged ≥80 increased by 46% (from 25,450 to 36,946) (Attachment 1). The age-standardized mortality rate (ASR) and the deaths per 100,000 in different age groups are shown separately in Table 2 (Tab. 2). Deaths/100,000 in total, and ASR decreased by 16% and 18%, respectively.

**Figure 2:**
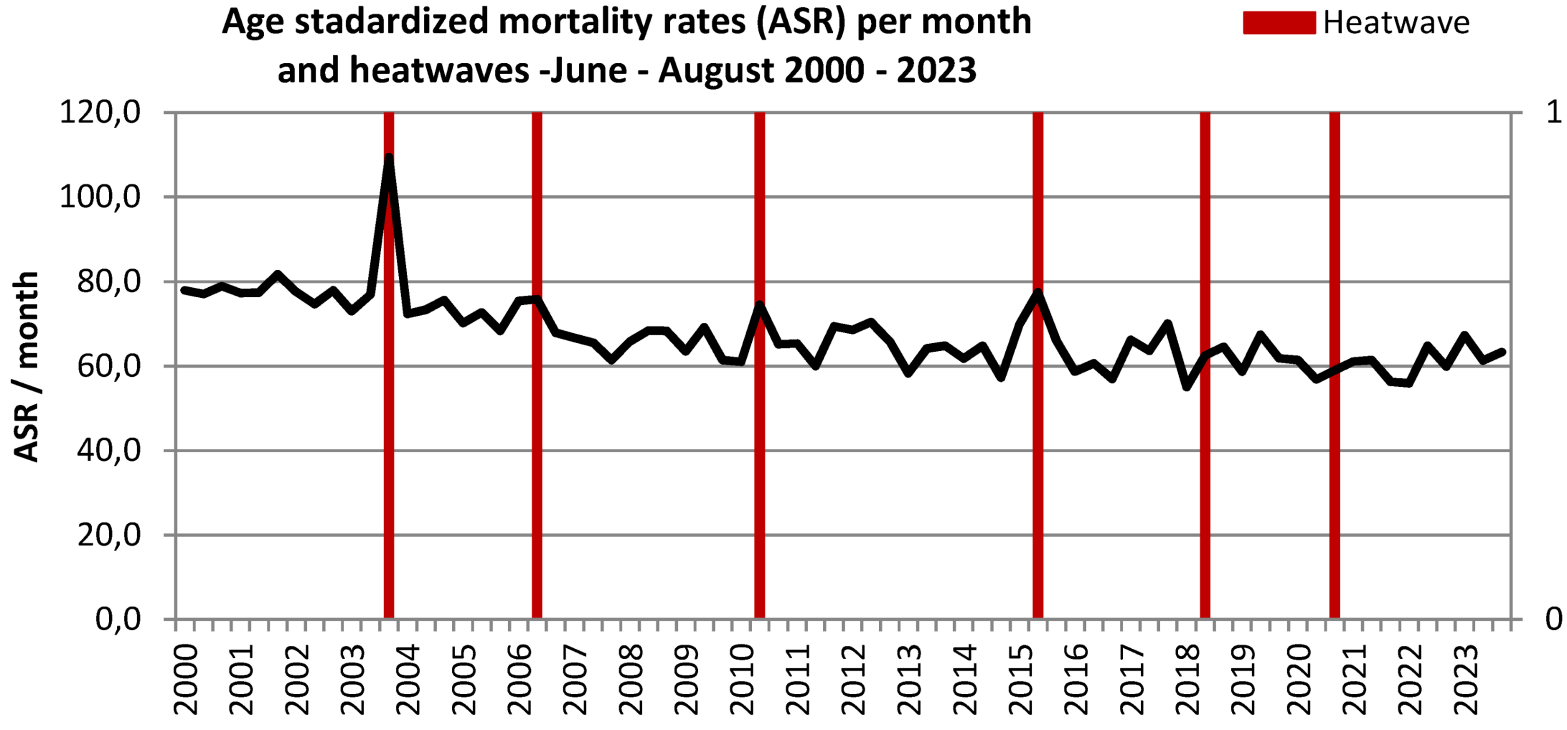
Age-standardized mortality rates (ASR) per month and heatwaves in Frankfurt am Main, Germany, June to August 2000–2023

**Figure 3:**
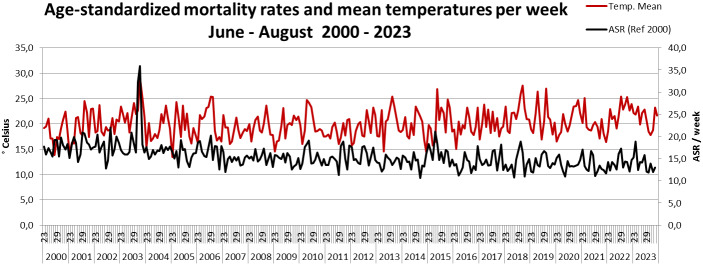
Age-standardized mortality rates (ASR) and mean temperatures per week in Frankfurt am Main, Germany, June to August 2000–2023

**Figure 4:**
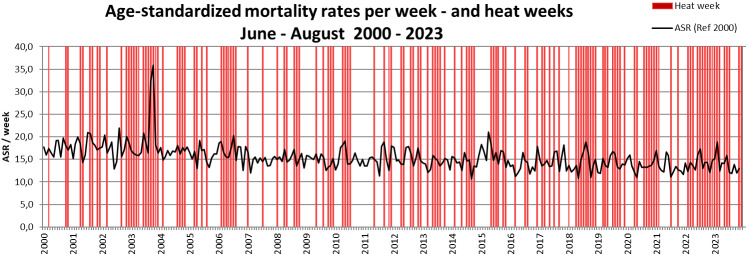
Age-standardized mortality rates (ASR) per week and heat weeks in Frankfurt am Main, Germany, June to August 2000–2023

The decreasing mortality is also evident in the ASR by month, despite exhibiting extreme values in August 2003 and during the July in 2006, 2010 and 2015 with documented heatwaves. In contrast, the heatwaves in 2018 and 2020 did not lead to significant excess mortality (Figure 2 (Fig. 2)). Figure 3 (Fig. 3) compares ASR with the weekly mean temperatures for calendar weeks 23 to 34 from 2000 to 2023. While the weekly mean temperatures show an increasing trend over the years, the ASR shows a tendency to decrease (Figure 3 (Fig. 3)). In Figure 4 (Fig. 4), the ASR per week is plotted in relation to the heat weeks. Despite a noticeable increase in heat weeks in recent years from 2018 onwards, no increase in ASR can be seen. All three charts (Figure 2 (Fig. 2), Figure 3 (Fig. 3), Figure 4 (Fig. 4)) show the highest increase in mortality by far in August 2003, with no such increase in mortality in any of the other heatwaves and heat weeks.

Incidence rates (i.e., deaths per 1 million person days) on heat days, heat warning days, days in heat weeks and heatwaves were compared to incidence rates on days without these exposures (Table 3 (Tab. 3), Table 4 (Tab. 4), Table 5 (Tab. 5), Table 6 (Tab. 6)). Across all years, 3.4 (95% confidence interval (CI): –1.6–8.5) more people per 1 million person days died on heat days than on non-heat days, which means a 16% increase in the incidence rates (Table 3 (Tab. 3)). Overall, there was a significant increase in incidence rates in 10 of the 24 years, a slight, non-significant increase in risk in 9 years and a slight decrease in mortality on heat days in 5 years.

The mean incidence rates were 1.9 (95% CI: –0.8–4.6), 2.8 (95% CI: –1.3–6.8), and 7.4 (95% CI: 2.1–12.7) deaths per 1 million person days higher during heat weeks, on days with heat warnings, and during heatwaves, respectively, than on days without these conditions (Table 4 (Tab. 4), Table 5 (Tab. 5), Table 6 (Tab. 6)). For all three conditions, strong differences were seen between the single years. In 2003, the increase in mortality during heatwaves was outstanding – the incidence rate was 23.2 deaths per 1 million person days higher during heatwaves than outside heatwaves (Table 6 (Tab. 6)). 

## Discussion

A trend towards higher summer temperatures as well as a trend towards an increase in heat weeks and heat days in Frankfurt am Main, Germany, was observed from 2000 to 2023. In the same period, age-standardized mortality rates in the summer months have decreased markedly. While the heatwaves in 2003 and 2015 still led to a sharp and significant increase in mortality in Frankfurt, no significant increase was evident in the later heatwaves in 2018 and 2020.

This is remarkable, because the hot summer of 2003 had the highest mean values for daily mean, maximum and minimum temperatures, the highest number of heat weeks and days of a heatwave, and the summer months of 2018 and 2022 showed comparable situations. In the summer months of all three years, the mean values of the daily mean temperature were at/above 22°C, the mean values of the daily maximum temperatures were >28°C and the daily minimum temperatures >15°C, the relative humidity <60% and the average precipitation <1 mm. In all three years, there were ≥10 heat weeks and a high number of heat days; in 2022, the highest value to date was reached with 22 heat days. Nevertheless, the population-related deaths in 2018 and 2022 show no remarkable anomalies – neither considering the summer months as a whole nor when considering the individual months or (heat) weeks.

In 2003, the number of deaths per 1 million person days was 16.0 higher on heat days than on non-heat days. The corresponding figures for 2018 and 2022 were considerably lower (3.8 and 2.3, respectively). This also applies to heat weeks. The corresponding figures were 6.9 for 2003, but only 1.7 and 2.5 for 2018 and 2022, respectively. 

The 2003 heatwave hit the population and the medical and care facilities in Frankfurt am Main completely unprepared. As a result, preventive measures – in particular information, training and continuing education for the health and care sectors – were established in the city starting in 2004, and the Frankfurt Climate Change Coordination Group was set up in 2006.

In its regular contacts with health and care facilities in the city, the public health department has repeatedly pointed out the need to be well prepared for periods of hot weather, and now has the impression that the problem has been recognized and appropriate precautions have been taken. The public is also increasingly aware of the importance of appropriate behavior in hot weather. However, there is no reliable data on the implementation of the recommended preventive measures. 

In a city ranking of 104 cities in Germany, based on strategies for adaptation as well as strategies for mitigation in 2020, Frankfurt was ranked 7^th^ in terms of climate mitigation, 4^th^ in terms of climate adaptation, and took second place when climate mitigation and adaptation were considered together. Frankfurt was thus classified in the top-rated cluster of climate policy leaders [25]. The assessment is based on the structures and targets surveyed, not (yet) on the implementation of measures.

In summary, our study in Frankfurt am Main not only showed a decrease in heat-related mortality in the population as a whole over the years but also a decrease in excess mortality during various heat periods (days, weeks, heatwave, heat warning), especially when comparing the years with very high heat stress and drought (2003, 2018, and 2022). Although it cannot be ruled out that other factors have also contributed to this phenomenon, such as decreasing air pollution, the trend indicates a certain adaptation of the population to the increasing heat. It will be interesting to see whether the observed trend continues in the coming years. 

Whether the development of heat-related mortality presented here describes a general trend or also reflects the intensive preventive measures that have been implemented in the city of Frankfurt for years cannot be answered by the present study. To this end, a comparative study should be carried out in various municipalities in the Rhine-Main region with different levels of commitment in dealing with the heat problem.

## Limitations and strengths

We refer to only one measuring station (the German National Meteorological Service measuring station in Frankfurt am Main) as an indicator for the entire urban area and the total population. This can only reflect the temperatures in the different residential areas of the city to a limited extent. However, this limitation also applies to all published studies, often to an even greater extent, as they are usually based on averaging over larger regions. e.g., entire federal states [3], [26], [27]. 

We only considered the summer months of June to August and the calendar weeks 23 to 34, comparable to our previous studies on heat-associated mortality and morbidity. In contrast, Winklmayer et al. included the summer half-year of April to September with the calendar weeks 15 to 40 in their studies of heat-related mortality [3], [27]. A further analysis (data not shown) of the situation in Frankfurt am Main from 2000 to 2023 showed that the period we selected comprises 97% of the heat days, 96% of the heat warning days and 86% of the heat weeks compared to the entire summer half-year. In view of these minor differences, we will continue to use our previous definition. 

Our study includes the deceased residents of Frankfurt and not all persons who died in Frankfurt. Many people who do not live in Frankfurt also die in the large maximum-care hospitals in Frankfurt; their share may well account for 30–40% of the documented deaths in Frankfurt. However, this cannot be related to a population, as it is not known how many patients from outside (region or tourists) are treated in Frankfurt hospitals. By limiting the calculation to deceased citizens of Frankfurt, it is possible to calculate age-standardized mortality rates and incidence rates per 1 million person days, thus enabling comparisons between years, even in a growing population. Without considering the strong population increase in Frankfurt from 2000 to 2023 (+25%) and in particular the increase in those age ≥80 years (+45%), the trend in heat-related mortality over the years would be strongly overestimated. This was also recently shown when looking at the excess mortality possibly associated with COVID-19 in Frankfurt am Main [28].

In the present study, we encompass four exposure indicators, i.e., heat days, heat warning days, days during heat weeks, or heatwaves on mortality, whereas in our previous studies we only examined the effects of heatwaves. Days with maximum temperatures of ≥32°C were defined heat days, as in our recent study on heat morbidity [21]. Because a general definition of heatwaves still does not exist [29], [30], [31], we retained the heatwave definition already published in our earlier studies (defined as at least 5 heat days with maximum ≥32°C in a row). With regard to the definition of the heat week, we refer to the work of An der Heiden et al. [26], who focussed on the population of Southwest Germany and published a temperature threshold close to 20°C daily mean temperature, above which mortality occurs. Here we define “heat week” as a week with a weekly mean temperature of ≥20°C. We also refer to heat warnings, which have been released by the German National Meteorological Service since 2005, differentiated according to the expected temperatures in individual regions through to urban and rural districts. The warnings are based on the concept of perceived temperature, i.e., on a physiological concept that considers not only the temperature, but also other weather parameters and nocturnal cooling [32], [33], [34]. Heat warnings are issued when a high heat load above 32°C perceived temperature is predicted and sufficient cooling of living spaces at night is no longer guaranteed [4]. When comparing the various exposure indicators, it should be noted that the heat day and heatwave models only take into account the maximum daily temperature, while the heat week and heat-warning models also reflect the night temperature; and the heat-warning model also includes other influencing factors. 

## Conclusion

Our study not only showed a decrease in heat-related mortality in the population as a whole over the years but also a decrease in excess mortality during various hot periods (day, week, heatwave, heat warning), especially when comparing the years with very high heat stress and drought (2003, 2018, and 2022). Even if it cannot be ruled out that other factors have also contributed to this trend, such as decreasing air pollution, this trend indicates a certain adaptation of the population to the increasing heat. It will be interesting to see whether the observed trend continues in the coming years.

## Notes

### Competing interests

The authors declare that they have no competing interests.

## Supplementary Material

Population development in Frankfurt am Main, Germany, 2000 to 2023

## Figures and Tables

**Table 1:**
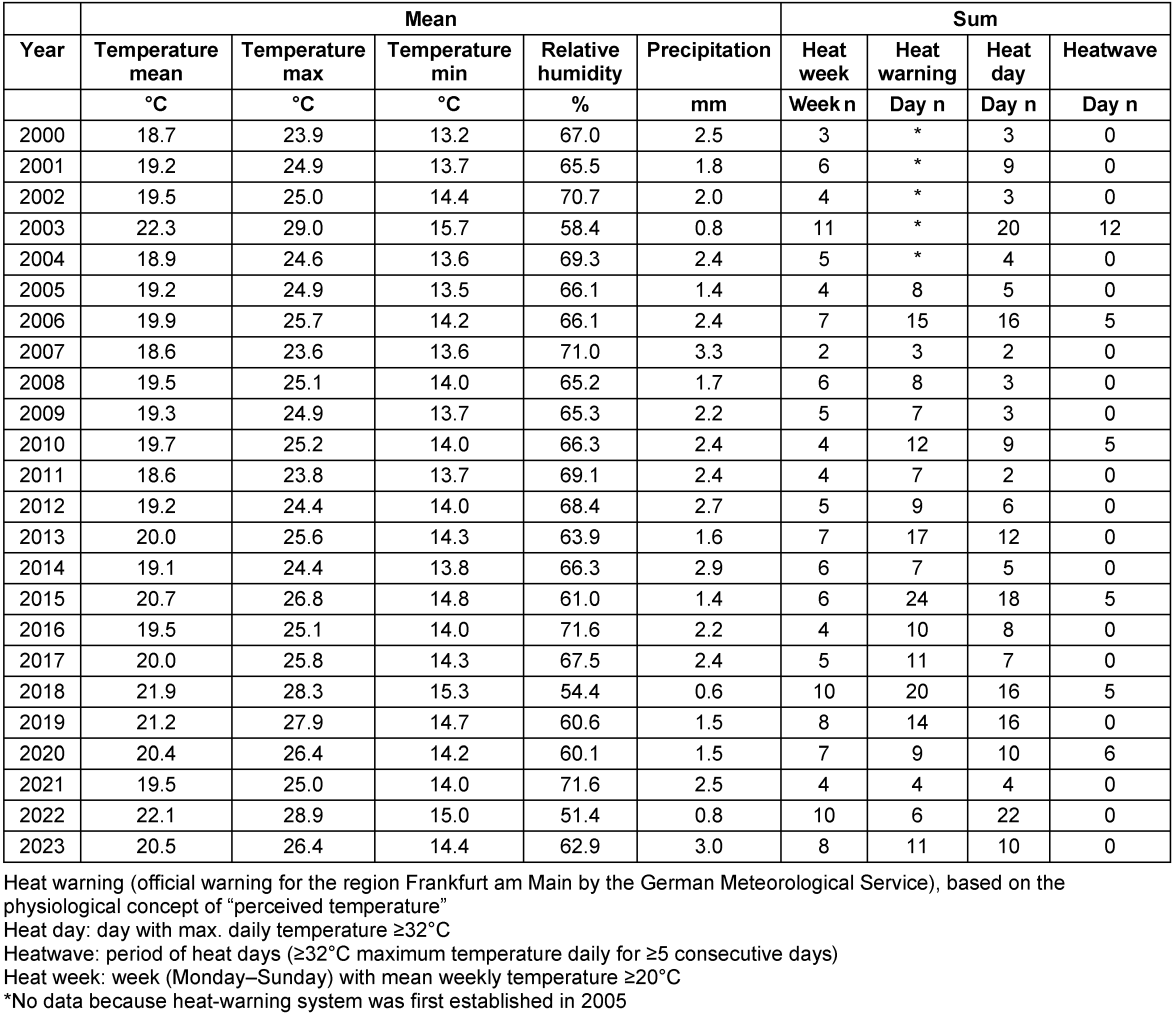
Meteorological data at the German National Meteorological Service measuring station in Frankfurt am Main, Germany, June to August 2000–2023

**Table 2:**
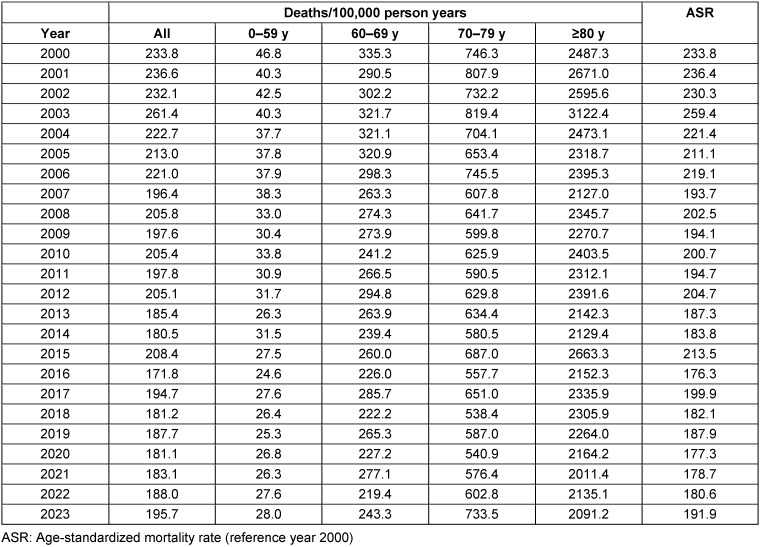
Deaths/100,000 person years in Frankfurt am Main, Germany, and age-standardized mortality rates (ASR) in the summer months of June to August 2000–2023 in Frankfurt am Main, Germany

**Table 3:**
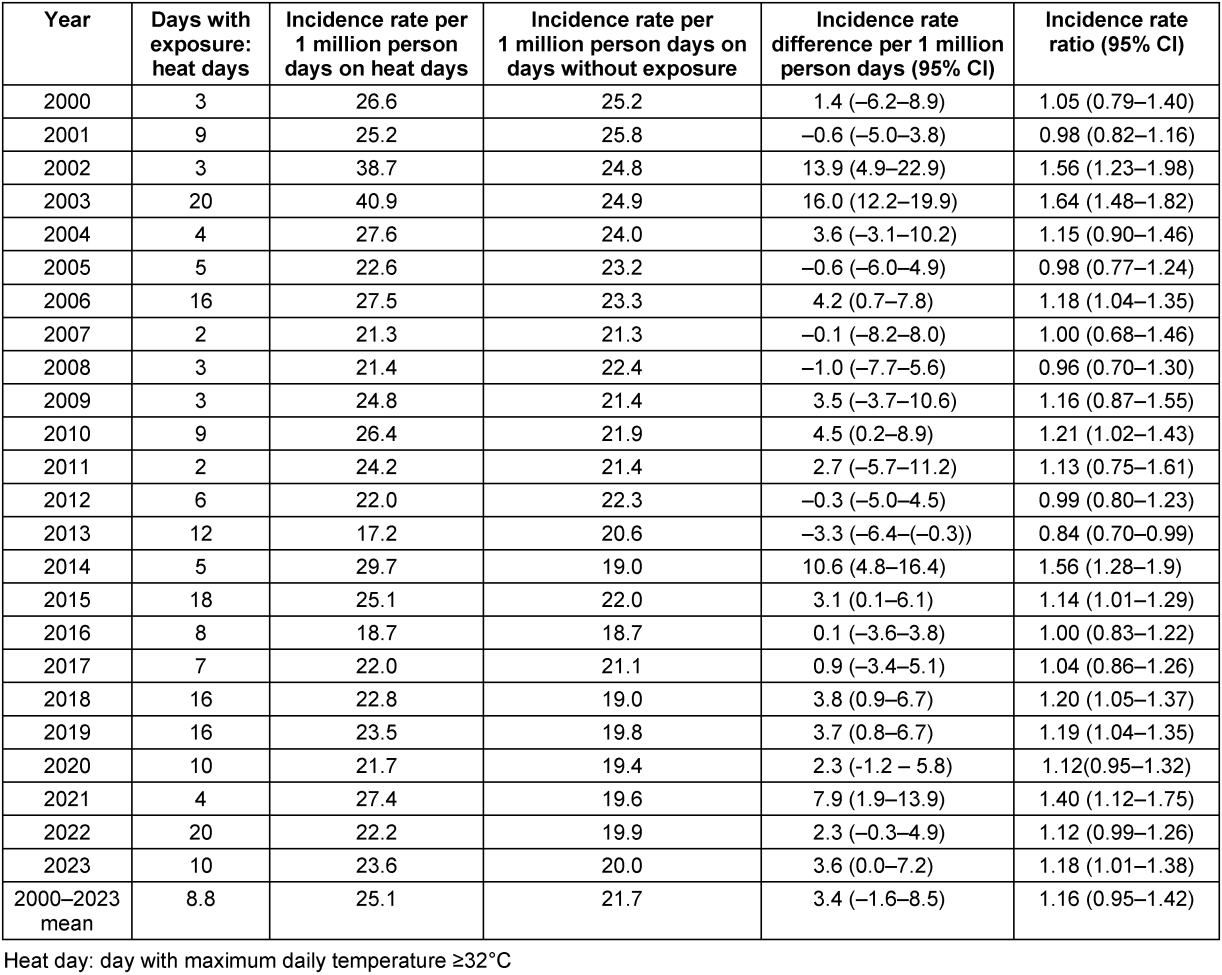
Comparison of incidence rates per 1 million person days on heat days and in periods outside heat days in Frankfurt am Main, Germany, June to August 2000–2023

**Table 4:**
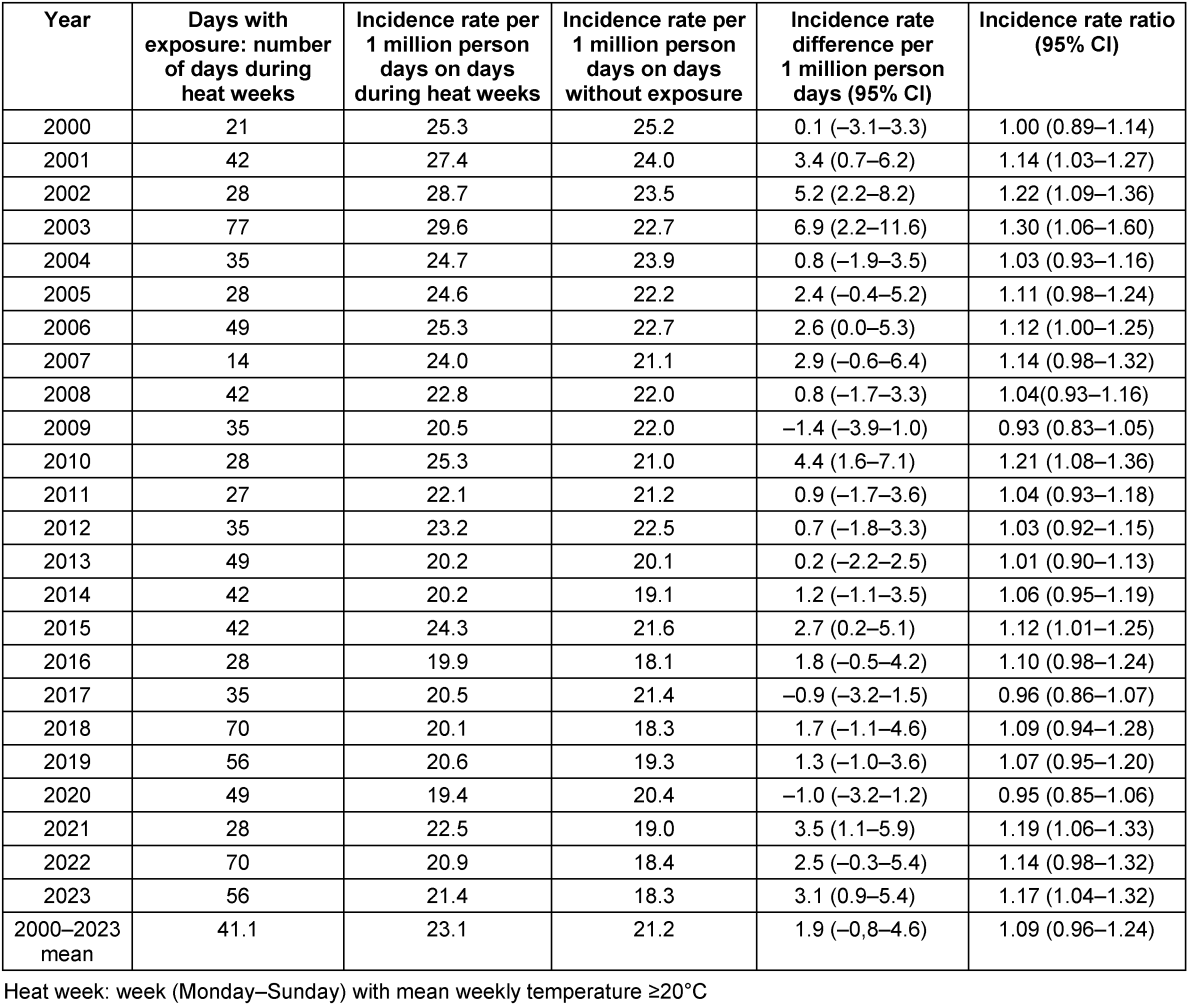
Comparison of incidence rates per 1 million person days during heat weeks and non-heat weeks in Frankfurt am Main, Germany, June to August 2000–2023

**Table 5:**
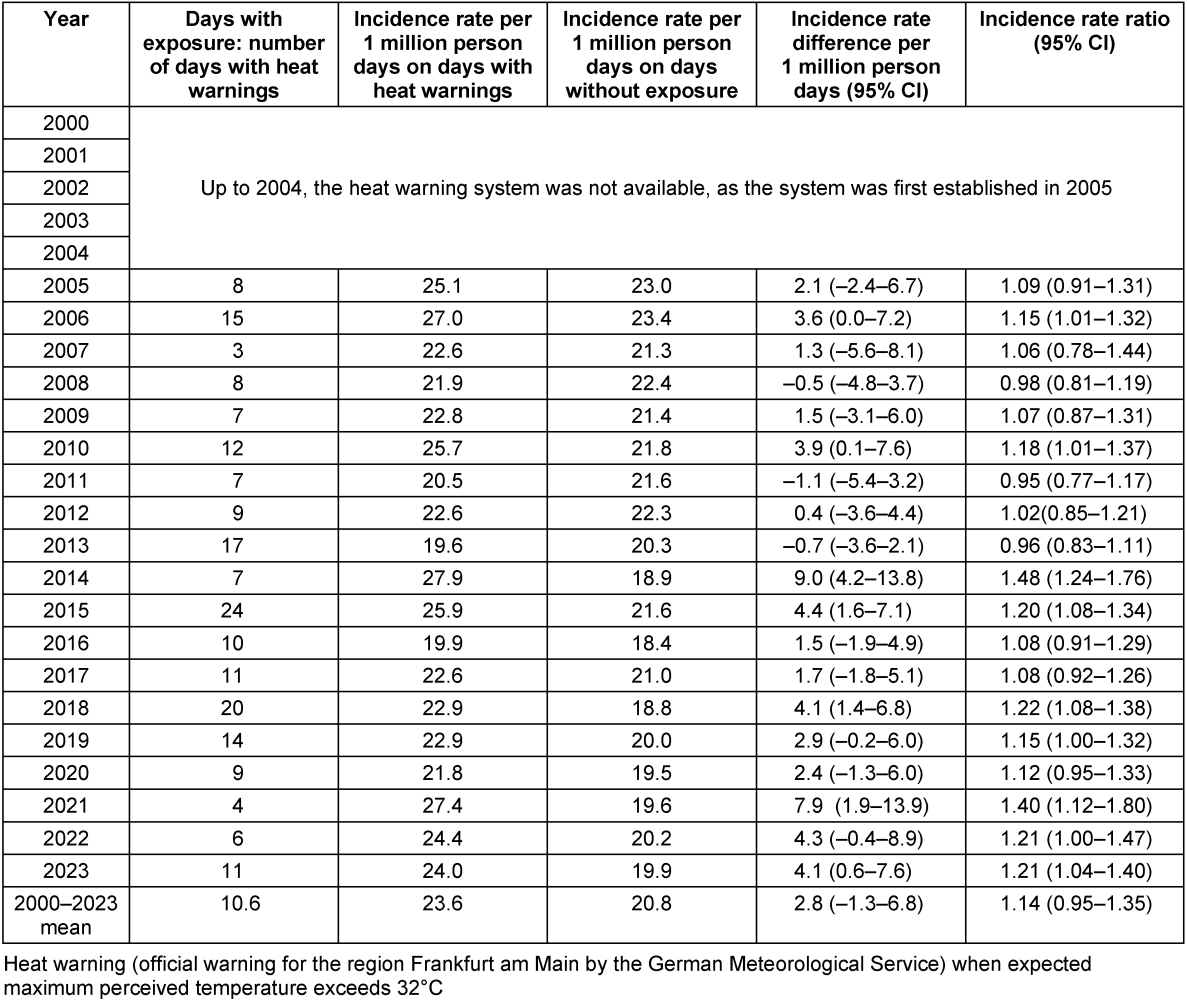
Comparison of incidence rates per 1 million person days on days with heat warnings and on days without heat warnings in Frankfurt am Main, Germany, June to August 2000–2023

**Table 6:**
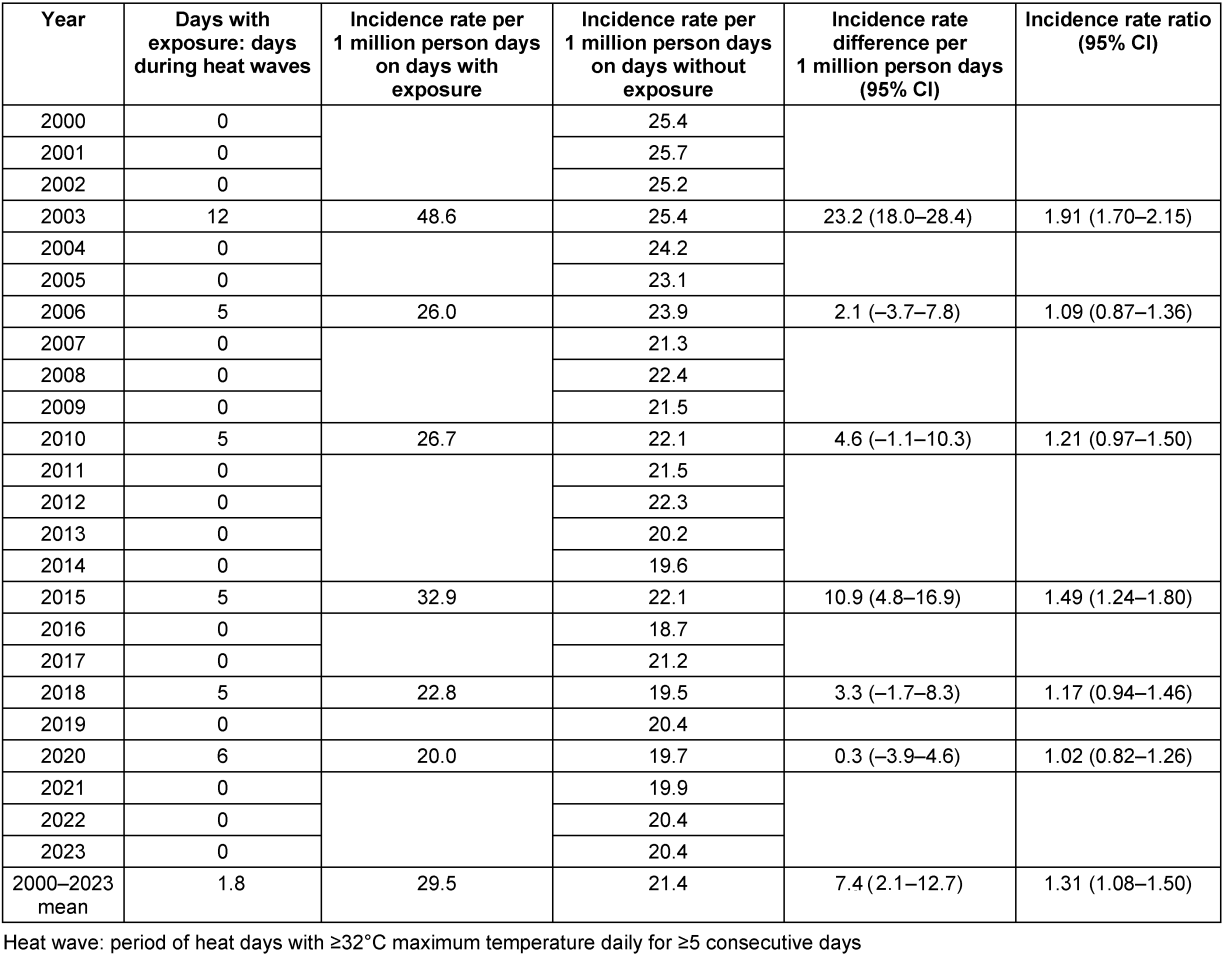
Comparison of Incidence rates per 1 million person days during heatwaves and on periods outside heatwaves in Frankfurt am Main, Germany, June to August 2000–2023
