# Supplementary material for: Heat-related mortality in Frankfurt am Main, Germany, from 2000 to 2023
Source: GMS Hyg Infect Control. 2024 Apr 30;19:Doc22. doi: 10.3205/dgkh000477 (PMC11099539; doi:10.3205/dgkh000477)
Supplement: Population development in Frankfurt am Main, Germany, 2000 to 2023 [file HIC-19-22-s-001.pdf]

Attachment 1: Population development in Frankfurt am Main, Germany, 2000 to 2023

Year	All	0–59 y	60–69 y	70–79 y	≥80 y
	n	n	n	n	n
2000	613,886	474,096	66,504	47,837	25,450
2001	622,212	481,452	67,823	46,543	26,395
2002	620,952	480,022	68,830	45,209	26,892
2003	622,905	481,665	69,934	44,180	27,127
2004	624,278	482,889	70,391	43,745	27,253
2005	627,815	486,560	69,492	44,075	27,688
2006	631,315	490,591	68,053	44,533	28,139
2007	634,476	493,687	67,220	45,079	28,491
2008	638,950	497,690	66,709	45,817	28,734
2009	644,802	502,859	66,080	47,018	28,846
2010	652,439	509,156	65,510	48,733	29,041
2011	661,751	517,269	64,913	50,463	29,108
2012	672,883	527,354	64,787	51,767	28,977
2013	686,017	539,153	65,168	52,802	28,894
2014	700,943	552,533	65,583	53,570	29,257
2015	716,515	566,723	66,551	53,279	29,963
2016	727,055	576,227	67,711	52,360	30,758
2017	735,359	583,114	68,604	51,919	31,723
2018	744,471	590,706	69,294	51,816	32,655
2019	753,211	597,607	70,110	51,617	33,878
2020	758,711	600,667	71,303	51,394	35,348
2021	756,237	596,528	72,532	50,834	36,343
2022	764,474	601,752	74,745	50,929	37,048
2023	767,434	603,021	76,462	50,987	36,964

2000–2021: Population data referring to 31st December were obtained from [24] and midyear populations were calculated as the average of the population figures on two consecutive years for 31st December. In 2022 and 2023, midyear populations were obtained directly from the Citizen's Office for Statistics and Elections, Frankfurt am Main, Germany.
